# Effects of aging and type 2 diabetes on resting and post occlusive hyperemia of the forearm; the impact of rosiglitazone

**DOI:** 10.1186/1472-6823-5-4

**Published:** 2005-03-24

**Authors:** Jerrold Petrofsky, Scott Lee, Maria Cuneo

**Affiliations:** 1 Physical Therapy Department, Loma Linda University, Loma Linda, USA; 2 School of Medicine, Loma Linda University, Loma Linda, USA

## Abstract

**Background:**

Both Diabetes and ageing are associated with reduced vascular endothelial function. The exact relationship between the 2 and any improvements from the insulin sensitizer rosiglitazone have not been explored.

**Methods:**

Thirty controls and sixteen subjects with type 2 diabetes participated in a series of experiments to examine the interrelationships between age, diabetes and endothelial cell function. In subjects with diabetes, the insulin sensitizer rosiglitazone (RSG), a drug also known to improve vascular function, was administered for 3 months to see how it altered these relationships. Resting forearm flows (RF) and blood flows after 4 min of vascular occlusion (PF) were measured as an index of endothelial cell function.

**Results:**

RF, measured by venous occlusion plethysmography, was negatively correlated to both age and diabetes. Administration of RSG for 3 months was associated with an increase in the blood flow response to venous occlusion so that it was not significantly different than that of age matched controls. Total PF in control subjects, compared to subjects with diabetes, averaged 56.58 +/- 12.57 and 13.6 +/- 8.01 cc/100 cc tissue per min respectively, and were significantly different (p < 0.01). After 3 months on RSG, differences between PF in the two groups were no longer evident.

**Conclusion:**

These studies suggest a different mechanism causing a reduction in vascular reactivity with aging and diabetes.

## Background

Normally, aging results in the natural senescence of multiple organ systems including the kidney [1], the autonomic nervous system [2], and the heart [3]. While numerous mechanisms are involved in age related changes in the body, one important factor contributing to decreased function is a reduction in nitric oxide production (a potent vasodilator) in tissues [4]. In addition, there is a reduction in beta adrenergic receptor sensitivity associated with the aging process [5], reducing the ability of the sympathetic nervous system to respond to stress.

Diabetes, similar to the aging process, can be associated with autonomic nervous system impairment including endothelial dysfunction and impairment of autonomic neurons [6,7]. This reduces function in both the sympathetic [8] and the parasympathetic nervous systems [9]. It can include loss of parasympathetic peripheral nerves and damage to autonomic ganglia, both of which are believed caused by vascular endothelial function [10,11]. Thus when the body is placed under stress, almost 30% of all people with diabetes show autonomic impairment. When autonomic stressors are combined, such as orthostatic changes and heat exposure together, nearly all patients with diabetes showed severe autonomic impairment [7].

The common mechanism for many of these changes with aging and diabetes is the inability of the blood vessels to adequately dilate. This leaves a predominant vasoconstrictor tone, causing inadequate blood supply to the skin and various organs of the body [12,13].

Endothelial cell function can be assessed by the change in blood flow after occlusion of the circulation [14]. Since the vasodilatation that results from local anoxia is entirely dependent on endothelial cell function and independent of the autonomic nervous system, this technique shows the effect of aging and diabetes only on vascular endothelial cells without the complication of involving other elements of the nervous system. The relationship between age, diabetes and vascular reactivity to occlusion should provide some evidence as to the interrelationships between these factors. This was one purpose of the present investigation.

Thiazolidinediones have been shown to increase blood flow in patients with type 2 diabetes by increasing nitric oxide production in vascular endothelial cells [28]. Further, administration of rosiglitazone, a member of this family (RSG), has been shown to increase resting forearm blood flows in both non diabetics and people with diabetes [15]. Thus, in the present investigation, a series of experiments was accomplished on patients with diabetes to assess the interrelationships between age, diabetes and RSG administration for 3 months to see if RSG will improve either the diabetes or age related loss in vascular reactivity to occlusion or both.

## Methods

### Subjects

The subjects with diabetes in this study were 8 men and 8 women, all with type 2 diabetes. The mean age, heights, BMI (Body Mass Index) and weights are shown in table 1 at the onset of the study. There were 15 on ACE inhibitors, 50% were on a statin, 3 were on beta- blockers and all but 1 were non smokers. Fifty percent were classified as having coronary artery disease and 50% had microalbuminuria. Sixty six percent had neuropathies, and 75% had retinopathies. Mean baseline values were: HbA1c: 8.8+/-2.4, Total cholesterol: 213+/-80, LDL: 125+/-80, HDL: 45+/-18, and Triglyceride: 275+/-274. Blood pressure was 132+/-38 systolic and 82+/-29 diastolic. Fourteen of the 16 were identified with hypertension.

**Table 1:** demographics of subjects with diabetes

	Number	Age (years)	height(cm)	weight(kg)	BMI	HBA1C
men	8					
mean		60.0	185.1	101.8	29.6	9.0
sd		19.9	12.5	24.2	5.7	2.8
women	8					
mean		63.8	161.9	83.2	31.1	8.4
sd		11.7	6.3	27.3	7.9	1.5
group mean	16	61.2	178.0	96.1	30.0	8.8
sd		10.2	15.6	26.7	6.3	2.4

Analysis of 3 month values revealed significantly lower HbA1c: 7.0 (P < .05) and lower trends in cholesterol and blood pressure: total cholesterol 206, LDL: 117, HDL: 42.9, and Triglyceride: 280. Blood pressure was 126+/-34 systolic and 72+/-22 diastolic. The reduction in both systolic and diastolic blood pressure was significant.

All medications were kept constant throughout the study.

The younger volunteers represented a healthier cohort than the patients with diabetes. None of the subjects had metabolic syndrome, and none smoked. They had a lower BMI, weight, and were about 5 years younger than the subjects with type 2 diabetes. None took any type of medication. Their average resting blood pressure was 121+/-34 systolic and 75+/-28 diastolic. For the control group, the BMI in the younger subjects (less than 45 years) was 23.2+/-6.1 while the older subjects (>45) averaged 27.9+/-5.3. Both younger and older groups of controls were all physically active faculty and staff at Loma Linda University, School of Allied Health.

### Measurements

Forearm blood flows – Forearm blood flows were measured by Whitney strain gauge plethysmography. Whitney strain gauge plethysmography is a technique of measuring limb blood flow by volume plethysmography, a full description is given elsewhere [14,16].

### Procedures

Muscle temperature varies in the forearm between 27 and 42 deg C. This is due to the fact that the forearm is a shell tissue and temperature varies to help gain or loose heat from the core of the body [17]. Limb tissue temperature varies with room temperature, clothing, body fat content, and the phase of the menstrual cycle [18]. Therefore, a thin person may have resting arm metabolism less than 20% of that of a person with a high body fat content due to the high Q10 of the tissues [19]. Therefore, to remove some of the variability in previous studies, a water bath was used to elevate all forearm temperatures to that of the core, 37 deg C. Subjects placed their arms in a bath heated to core body temperature of 37 deg C with the arm held dependant and the elbow at an angle of 90 degrees such that their arms were submerged to the belly of the biceps muscle. The bath was well stirred. After 15 minutes, resting arm flows were recorded. A 4 min period of arterial occlusion was then induced by a cuff under the axilla and inflated to 200 mmHg was then used. The cuff was then released and blood flow was measured for another 2 minutes. All experiments were repeated on controls and subjects with diabetes prior to administration, after 2 and 4 weeks, and 3 months on RSG. Measurements on the control subjects were repeated at 1 and 3 months.

### Statistical analysis

Statistical analysis involved the calculation of means, standard deviations, T tests, and analysis of variance (ANOVA). The level of significance was p < 0.05. All data in "results" is expressed ± the standard deviation. Regression lines were calculated by the method of least squares.

## Results

**Figure 1:**
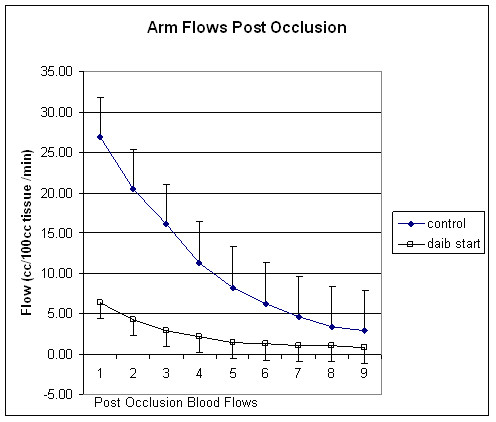
This figure shows the blood flow recorded for 2 minutes following the release of an arterial occlusion cuff on the brachial artery in control subjects (diamonds) and subjects with diabetes (squares). Illustrated here are the average results for all subjects in each group +/- standard deviation. Flows are expressed in cc/100 ml muscle per minute and the time scale on the bottom is the flow number. Flows are recorded every 12 seconds starting at 3 seconds post occlusion.

**Figure 2:**
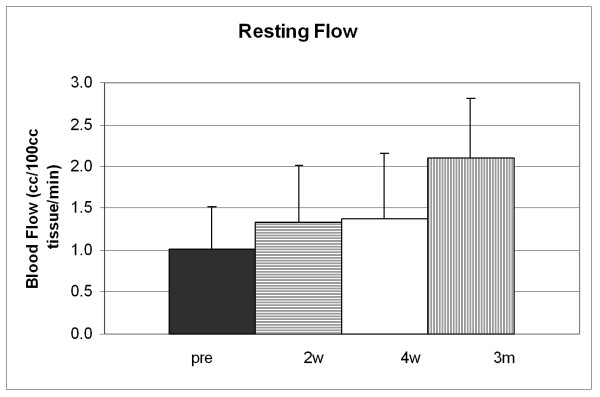
This figure shows the blood flow in the forearm of the subjects with diabetes at rest (cc/ 100 ml tissue/minute) before taking rosiglitazone (pre) and at two weeks (2 w), four weeks (4 w), and three months (3 m) taking 4 ml per day of Rosiglitazone. All data is shown with the appropriate standard deviation.

**Figure 3:**
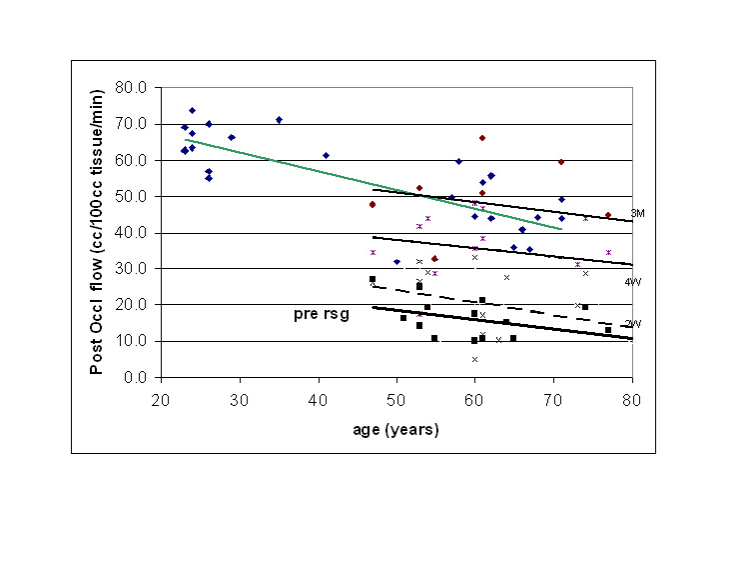
This figure illustrates the excess flow above rest during a two minute period after the release of an occlusion cuff on the brachial artery of control subjects and subjects with diabetes. Individual data points are shown on control subjects and subjects with diabetes prior to initiation of a three month administration of rosiglitazone (pre rsg) and after 2 weeks (2 w), four weeks (4 w), and three months (3 m). Data is plotted in relationship to the age of the subject. The regression lines are calculated by the method of least squares. On the control subjects the regression line was flow = -0.518 age + 77.78. Prior to administration of rosiglitazone of the subjects with diabetes the regression equation was flow = -0.253 age + 31.01. After two weeks of administration of rosiglitazone the regression equation was blood flow = -0.348 age + 41.6. After 4 weeks of rosiglitazone the regression equation was blood flow = -0.243 age + 50.27. Finally after 3 months of rosiglitazone was post occlusion flow = -0.274 age + 64.94

The results of the experiments are shown in figures 1, 2, 3. Figure 1 illustrates the blood flow in the control subjects and subjects with diabetes prior to administration of RSG after 4 min of vascular occlusion. Prior to occlusion, the average blood flow in the control subjects was 2.24 +/- 0.64 cc/100 cc tissue/min. After occlusion, for the control subjects, blood flow peaked at 26.8 +/- 4.86 cc/100 ml tissue/min for the first flow approximately 3 seconds after vascular occlusion; 2 minutes post occlusion, the flow was back to the initial resting flow. In contrast, for the subjects with diabetes, blood flow at rest was only 1.0 +/- 1.02 cc/100 ml tissue/min. After 4 minutes of occlusion, the peak flow only averaged 6.4 +/- 2.82 cc/100 ml tissue/min and after 1 minute post occlusion, flow was 1.5 +/- 1.42 cc/100 ml tissue/min. The total blood flow response to occlusion was significantly less in the subjects with diabetes (p < 0.01). Since the duration and magnitude of the blood flow response to occlusion were different in the subjects with diabetes compared to control subjects, a separate calculation was made, the excess flow. This was accomplished by calculating the total blood flow above the normal resting flow observed in the 2 minutes following vascular occlusion. For the control subjects, the average excess flow was 56.58 +/- 12.57 cc/100 ml tissue of blood whereas for the subjects with diabetes the excess flow was 13.6 +/- 8.01 cc/100 cc tissue resulting from the occlusion, the data in the control group was significantly higher than for the group with diabetes (p < 0.01).

After subjects were placed on 4 mg RSG for 3 months, resting blood flow increased. Resting flows in subjects with diabetes, when compared to the control subjects, were significantly less prior to administration of RSG (p < 0.01) and after 2 weeks and 4 weeks this difference was preserved (p < 0.05). However, after 3 months, while the resting flows were still numerically less in the subjects with diabetes, due to the large variance and small number of subjects, there was no statistical difference in resting blood flows between controls and subjects with diabetes (p > 0.05). The total excess flows increased before and after 2 weeks, 4 weeks and after 3 month administration with RSG. Blood flows were 13.06+/-8.01, 19.01 +/-8.00, 35.2+/-13.4 and 42.08 +/-20.08 cc/100 cc tissue. Before and at 3 months, the flows, when compared to the controls, were significantly less (p < 0.05).

Since aging has a known effect on resting and exercising blood flow, the data in figure 3 has been plotted in relationship to age. For the control subjects, for example, as shown at the top of the figure, there was a linear decrease in post occlusion flows (as well as resting flows) associated with aging. The regression line through the data points in fig 3 showed that for every year a subject aged, they lost 0.51 cc of blood flow per 100 g tissue per minute in other words, a loss of approximately 5 cc in post occlusive reactive hyperemia for every 10 years increase in age.

For the subjects with diabetes, prior to taking RSG, there was also an age related loss in excess flow as shown in the figure. The slopes of the lines between the non-diabetic and diabetic subjects prior to taking RSG were not statistically different from each other (p < 0.01). Thus for subjects with diabetes, there was a similar reduction in post occlusive hyperemia with aging to a greater extent than in control subjects. After 2 and 4 weeks on RSG, there was still the same aging effect on the reduction of post occlusive flow however; difference between the groups was reduced. After 3 months there was no significant difference in flows in controls and patients with diabetes when related to age (p > 0.05).

For the controls, the correlation between age and excess flow was -0.56, a significant correlation (p < 0.01). With an R2 of 0.31, 30% of the change in flow after occlusion could be accounted for by age alone. In the subjects with diabetes, the correlation between age and flow was 0.71 with an R2 of .49 before RSG. Two weeks after RSG the correlation was 0.61, after 4 weeks was 0.57. After 3 months it was 0.45. Thus, diabetes seems to potentate ageing. Before RSG, 20% more of the variability in flows with age was observed. After 3 months on RSG, the contribution of ageing to blood flow was the same.

## Discussion

Both aging and diabetes are associated with a loss in function of the cardiovascular system. One commonly reported factor in both is a reduction in the ability of the peripheral vasculature to vasodialate [1,2,20]. Normally, with the arm at rest, there is always both tonic vasoconstrictor and vasodilator tone to the vascular endothelial cells. In the skin, for example, there is a slow tonic release of norepinephrine, a vasoconstrictor, and acetylcholine, a vasodilator [21-23]. If there is an increase in vasoconstrictor activity, then vasoconstriction predominates and if more vasodilator activity is present, then vasodilatation predominates [24]. Thus, even reflex changes in blood flow can be modulated up or down by central sympathetic constrictor or dilator activity [24].

In the present investigation, resting blood flow and the blood flow after vascular occlusion was reduced with aging and diabetes. These results agree with that shown by others [15,25]. At first examination, it would appear that a similar mechanism might be involved in reducing vascular reactivity with age and diabetes. Certainly, in the present investigation, older subjects with diabetes had a greater reduction in resting flows and post ischemic flows than was seen in younger subjects. A simple loss in the ability to vasodilate would explain both the reduced post ischemic flows and the shorter duration of the flows. If vasodilatation were impaired, then blood flows would be less after ischemia and, when flows began to return to pre ischemic levels, the dominant vasoconstriction would shorten the hyperemia as observed here. But the hyperemia was not shortened with age for control subjects, only subjects with diabetes suggesting different mechanisms causing the reduction in blood flow between age and diabetes.

Part of the mechanism for reduced resting and post ischemic blood flow with both aging and diabetes has been linked to a reduction of the vascular endothelial cells ability to produce nitric oxide, a potent vasodilator substance [4]. This may be due to a defect in nitric oxide synthesis, decreased nitric oxide sensitivity or reduced availability of l-arginine, the precursor of nitric oxide. Nitric oxide is released by vascular endothelial cells in response to a variety of stimuli including sympathetic vasodilator nerves [4]. But here is where the similarity may end.

Data presented here make it unlikely that ageing and diabetes effect endothelial function by a similar mechanism. In the graph in figure 3, the relationship between age and cardiovascular reactivity (excess flows) was linear. For the subjects with diabetes, there was no statistical difference in the slopes but the intercept was very different; 77.78 cc for the control subjects and 31.01 cc for the subjects with diabetes. Simply looking at these 2 groups of subjects, it might be inferred that diabetes potentiated the loss in endothelial function with age. And yet, after 3 months on RSG, there was no statistical effect of diabetes alone on vascular endothelial function but ageing still had a pronounced effect. If a similar mechanism were involved, i.e. loss of NO in both, it seems too coincidental that the curves remained parallel relating age to endothelial function in subjects with diabetes and after 3 months on RSG; the data from the diabetic subjects would exactly match the controls.

It seems more likely that 2 separate mechanisms are involved. While there is a loss in nitric oxide production with age, it is not clear if this is the sole culprit in causing a loss of endothelial function with age. The studies on rosiglitazone also point to this possibility. Recent studies show at least 2 other mechanisms associated with ageing. First, there is a loss in beta-adrenergic receptor sensitivity with ageing due to structural changes in the receptor making it insensitive to catacholamines [5]. Since the response to occlusion is not mediated by sympathetic nerves, this probably plays little role here. But in a recent study [26], data was presented which shows that nitric oxide may not be the predominant factor in controlling skin circulation after occlusion. These investigators found that the relative NO-dependent portion of cutaneous active vasodilatation accounted for 23% of vasodilatation in young subjects. Other factors were responsible for most of cutaneous vasodilatation. Even in older subjects, nitric oxide contributed to only 60% of the vasodilatation. Thus, other mechanisms were involved and possibly other relaxation factors altering smooth muscle tone in the vascular beds [27].

Rosiglitazone is a PPAR gamma ligand that has been shown to increase skin circulation [15,28,29] by increasing nitric oxide production [28]. The fact that nitric oxide increases with administration of rosiglitazone and yet the aging effect on endothelial function was not altered supports the concept of another mechanism involved here. The improvement in glucose control could also contribute to the improvement in endothelial function seen in this experiment. Finally, the use of Beta blockers in a few of the subjects and physical activity may have had some effect on these results. Hypertension, in itself may have caused endothelial damage here. However, there was no difference in the group of subjects with beta blockers and the smokes in their response to RSG. While it is well accepted that the response to vascular occlusion is a means of examining endothelial function [30], and that nitric oxide production is reduced in diabetes [31] and with ageing in vascular endothelial cells [32], the exact mechanism of the damage, e.g. nitric oxide, prostaglandins etc can not be fully elucidated without additional studies. More detailed studies are needed to understand these mechanisms.

## Conclusion

**1. **Aging is associated with a reduction in both resting forearm blood flows and post ischemic blood flows.

**2. **Diabetes further exacerbates the cardiovascular damage, reducing vascular endothelial cell reactivity.

**3. **Administration of RSG and improvement of glucose control appear to reverse the damage to vascular endothelial cells associated with diabetes but not aging.

**4. **The reduction in endothelial cell reactivity to occlusions with aging and diabetes are probably due to different mechanism.

## Abbreviations

RF- Resting forearm blood flows

RSG- Rosiglitazone

PF- Post occlusion blood flows

## Competing interests

The author(s) declare that they have no competing interests.

## Authors' contributions

Dr Jerrold Petrofsky developed the studies, collected data, analyzed the data and drafted the manuscript. Dr Scott Lee developed the studies, recruited subjects and helped draft the manuscript. Miss Cúneo collected data and analyzed data and helped draft the manuscript. All authors read and approved the final manuscript.

**Table 2:** Demographics of Control Subjects

	number		Age (years)	Height (cm)	weight (kg)	BMI
men	19	mean	54.0	176.2	86.3	24.5
		sd	18.2	9.7	16.7	30
women	11	mean	57	168.1	80.1	28.3
		sd	19.1	11.4	16.7	4.7
group	30	mean	55.2	174.2	84.2	26.4
		sd	22.3	17.2	22.3	5.7

## Pre-publication history

The pre-publication history for this paper can be accessed here:



## References

[B1] Fagard R, Thijs L, Amery A (1993). Age and the Homodynamic Response to Posture and Exercise. Am J Geriatr Cardiol.

[B2] Cybulski G, Niewiadomski W (2003). Influence of age on the immediate heart rate response to the active orthostatic test. J Physiol Pharmacol.

[B3] Rzeczuch K, Jagielski D, Kolodziej A, Kaczmarek A, Mielnik M, Banasiak W, Ponikowski P (2003). Coronary collateral circulation is less developed when ischaemic heart disease coexists with diabetes. Kardiol Pol.

[B4] Stadler K, Jenei V, von Bolcshazy G, Somogyi A, Jakus J (2003). Increased nitric oxide levels as an early sign of premature aging in diabetes. Free Radic Biol Med.

[B5] Schutzer WE, Mader SL (2003). Age-related changes in vascular adrenergic signaling: clinical and mechanistic implications. Ageing Res Rev.

[B6] Accurso V, Shamsuzzaman AS, Somers VK (2001). Rhythms, rhymes, and reasons – spectral oscillations in neural cardiovascular control. Auton Neurosci.

[B7] Petrofsky JS, Besonis C, Rivera D, Schwab E, Lee S (2003). Heat Tolerance in patients with diabetes. J Appl Research.

[B8] Sagliocco L, Sartucci F, Giampietro O, Murri L (1999). Amplitude loss of electrically and magnetically evoked sympathetic skin responses in early stages of type 1 (insulin-dependent) diabetes mellitus without signs of dysautonomia. Clin Auton Res.

[B9] Ewing DJ, Boland O, Neilson JM, Cho CG, Clarke BF (1991). Autonomic neuropathy, QT interval lengthening, and unexpected deaths in male diabetic patients. Diabetologia.

[B10] Shaw R (1977). Neuroendocrine control of sweat glands. J Invest Dermatol.

[B11] Fealey RD, Low PA, Thomas JE (1989). Thermoregulatory sweating abnormalities in diabetes mellitus. Mayo Clin Proc.

[B12] Stansberry KB, Peppard HR, Babyak LM, Popp G, McNitt PM, Vinik AI (1999). Primary nociceptive afferents mediate the blood flow dysfunction in non-glabrous (hairy) skin of type 2 diabetes: a new model for the pathogenesis of microvascular dysfunction. Diabetes Care.

[B13] Rendell M, Bergman T, O'Donnell G, Drobny E, Borgos J, Bonner RF (1989). Microvascular blood flow, volume, and velocity measured by laser Doppler techniques in IDDM. Diabetes.

[B14] Petrofsky JS, Besonis C, Rivera D, Schwab E, Lee S (2003). Does Local Heating Really Help Diabetic Patients Increase Circulation. J Orthop Neuro Surg.

[B15] Petrofsky JS, Bweir Sm, Lee S, Libarona M (2004). Rosiglitazone Improves Age Related Reductions in Forearm Resting Flows and Endothelial Dysfunction Observed in Type 2 Diabetes. Diabetes.

[B16] Whitney RJ (1953). The measurement of volume changes in human limbs. J Physiol.

[B17] Petrofsky JS, Le Donne D, Reinhart JS, Lind AR (1981). The influence of the menstrual cycle on blood flow through muscle during isometric contractions. Ohio J Sci.

[B18] Petrofsky JS, Lind AR (1975). The relationship of body fat content to deep muscle temperature and isometric endurance in man. Clin Sci Mol Med.

[B19] Wang G, Kawai M (2001). Effect of temperature on elementary steps of the cross-bridge cycle in rabbit soleus slow-twitch muscle fibers. J Physiol.

[B20] Adachi T, Kawamura M, Owada M, Hiramori K (2001). Effect of age on renal functional and orthostatic vascular response in healthy men. Clin Exp Pharmacol Physiol.

[B21] Huber KH, Rexroth W, Werle E, Koeth T, Weicker H, Hild R (1991). Sympathetic neuronal activity in diabetic and non-diabetic subjects with peripheral arterial occlusive disease. Klin Wochenschr.

[B22] Koch DW, Leuenberger UA, Proctor DN (2003). Augmented leg vasoconstriction in dynamically exercising older men during acute sympathetic stimulation. J Physiol.

[B23] Lacolley PJ, Lewis SJ, Brody MJ (1991). Role of sympathetic nerve activity in the generation of vascular nitric oxide in urethane-anesthetized rats. Hypertension.

[B24] Hornyak ME, Naver HK, Rydenhag B, Wallin BG (1990). Sympathetic activity influences the vascular axon reflex in the skin. Acta Physiol Scand.

[B25] Walker KZ, Piers LS, Putt RS, Jones JA, O'Dea K (1999). Effects of regular walking on cardiovascular risk factors and body composition in normoglycemic women and women with type 2 diabetes. Diabetes Care.

[B26] Holowatz LA, Houghton BL, Wong BJ, Wilkins BW, Harding AW, Kenney WL, Minson CT (2003). Nitric oxide and attenuated reflex cutaneous vasodilation in aged skin. Am J Physiol Heart Circ Physiol.

[B27] Jagren C, Gazelius B, Ihrman-Sandal C, Lindblad LE, Ostergren J (2002). Skin microvascular dilatation response to acetylcholine and sodium nitroprusside in peripheral arterial disease. Clin Physiol Funct Imaging.

[B28] Vinik AI, Stansberry KB, Barlow PM (2003). Rosiglitazone treatment increases nitric oxide production in human peripheral skin: a controlled clinical trial in patients with type 2 diabetes mellitus. J Diabetes complications.

[B29] Pistrosch F, Passauer J, Fischer S, Fuecker K, Hanefeld M, Gross P (2004). In Type 2 Diabetes, Rosiglitazone Therapy for Insulin Resistance Ameliorates Endothelial Dysfunction Independent of Glucose Control. Diabetes Care.

[B30] Faulx MD, Wright AT, Hoit BD (2003). Detection of endothelial dysfunction with brachial artery ultrasound scanning. Am Heart J.

[B31] Calver A, Collier J, Vallance P (1992). Inhibition and stimulation of nitric oxide synthesis in the human forearm arterial bed of patients with insulin-dependent diabetes. J Clin Invest.

[B32] Smith AR, Hagen TM (2003). Vascular endothelial dysfunction in aging: loss of Akt-dependent endothelial nitric oxide synthase phosphorylation and partial restoration by (R)-alpha-lipoic acid. Biochem Soc Trans.

